# The Diagnostic Accuracy and Clinical Utility of Three Noninvasive Models for Predicting Liver Fibrosis in Patients with HBV Infection

**DOI:** 10.1371/journal.pone.0152757

**Published:** 2016-04-06

**Authors:** Zhiqiao Zhang, Gongsui Wang, Kaifu Kang, Guobiao Wu, Peng Wang

**Affiliations:** 1 Department of Infectious Diseases, The Shunde First People’s Hospital, Shunde, Guangdong, China; 2 Department of Pathology, The Shunde First People’s Hospital, Shunde, Guangdong, China; Yonsei University College of Medicine, REPUBLIC OF KOREA

## Abstract

**Aim:**

To evaluate the diagnostic accuracy and clinical utility of the fibrosis index based on the four factors (FIB-4), aspartate aminotransferase -to-platelet ratio index (APRI), and aspartate aminotransferase–alanine aminotransferase ratio index (AAR) for predicting liver fibrosis in patients with HBV infection.

**Methods:**

From January 2006 to December 2010,a total of 1543 consecutive chronic hepatitis B(CHB) patients who underwent liver biopsies were enrolled. FIB-4,APRI, and AAR were calculated.The areas under the receiver-operating characteristic curves (AUROCs) were calculated to assess the diagnostic accuracy of these models.The AUROCs of these models were compared by DeLong’s test.For further comparisons in different studies,the AUROCs were adjusted to conduct Adjusted AUROCs(ADjAUROCs) according to the prevalence of fibrosis stages using the difference between advanced and nonadvanced fibrosis (DANA).

**Results:**

For prediction of significant fibrosis,severe fibrosis,and cirrhosis,the AUROCs of FIB-4 were 0.646(ADjAUROC 0.717),0.670(ADjAUROC 0.741), and 0.715(ADjAUROC 0.786) respectively;whereas it were 0.656(ADjAUROC 0.727),0.653(ADjAUROC 0.724) and 0.639(ADjAUROC 0.710) for APRI, 0.498(ADjAUROC 0.569),0.548(ADjAUROC 0.619) and 0.573(ADjAUROC 0.644) for AAR. The further comparisons demonstrated that there were no significant differences of AUROCs between FIB-4 and APRI in predicting significant and severe fibrosis(*P* > 0.05),while FIB-4 was superior to APRI in predicting cirrhosis(*P* < 0.001). Further subgroup analysis demonstrated that the diagnostic accuracy of FIB-4 and APRI in patients with normal alanine aminotransferase(ALT) were higher than that in patients with elevated ALT.

**Conclusions:**

The results demonstrated that FIB-4 and APRI are useful for diagnosis of fibrosis. FIB-4 and APRI have similar diagnostic accuracy in predicting significant and severe fibrosis,while FIB-4 is superior to APRI in predicting cirrhosis. The clinical utility of FIB-4 and APRI for fibrosis need further external validation in a large population before it was used for prediction of fibrosis in patients with HBV infection.

## Introduction

Hepatitis B virus (HBV) infection affects 350 million individuals and there are almost one million people died for HBV-related liver diseases every year[1]. Liver biopsy is still the gold standard for assessing hepatic fibrosis in patients with HBV infection. However, liver biopsy is limited by invasiveness and susceptibility of this technique to sampling error[2,3]. Magnetic Resonance Imaging (MRI), Computed Tomography (CT),and transient elastography(TE) have a better diagnostic value in detecting of hepatic fibrosis. However,these imaging examinations are limited by the high cost and not readily available in most hospitals. From the perspective of cost-effectiveness and clinical practice, an ideal diagnostic method for assessment of liver fibrosis should be a simple, noninvasive,inexpensive, readily available, and easier practical test. Therefore, FIB-4,APRI,and AAR had been suggested to evaluate the liver fibrosis[4–6]. However,the conclusions of these previous studies were controversial and their clinical utility for fibrosis in patients with HBV infection were uncertain[7–9].

Therefore,we performed this retrospective study to evaluate diagnostic accuracy and clinical utility of FIB-4, APRI,and AAR for predicting liver fibrosis in hepatitis B virus-infected patients.

## Materials and Methods

### Patients

Between January 2006 to December 2010, 1620 consecutive patients who had been diagnosed with HBV infection and had undergone a liver biopsy in department of infectious diseases of Shunde First People’s Hospital. The Patients were enrolled based on the following criteria: chronic hepatitis B(CHB) defined as hepatitis B surface antigen (HBsAg) positivity for more than 6 months; detectable HBV-DNA with a level >10^3^ copies/ml. The exclusion criteria were as follows: liver cancer or co-infection with hepatitis C virus, hepatitis D virus or human immunodeficiency virus; autoimmune liver diseases suah as autoimmune hepatitis, primary biliary cirrhosis, and primary sclerosing cholangitis; alcohol ingestion in excess of 20 g/day;hereditary and metabolic liver diseases suah as Wilson’s disease, hemochromatosis, and α-1-antitrypsin deficiency.

Therefore, there were 77 patients excluded from the study according to above criteria. There were no significant differences in terms of demographic and clinical parameters between patients included and excluded (data not shown).Finally, a total of 1543 patients (1182 males and 361 females) were recruited into the study. The written consent was obtained from patients before inclusion.The study was approved by the ethics committee of the Shunde First People’s Hospital. All clinical investigation were conducted according to the principles expressed in the Declaration of Helsinki.

### Liver biopsy

Liver biopsies were performed by two experienced physicians using a 16-gauge needle(16G biopsy Menghini’s needle, ShangHai). A minimum of 1.5 cm of liver tissue with at least 7 portal tracts was required for diagnosis.The specimens were fixed, paraffin-embedded and stained with haematoxylin and eosin (HE). Histological grading of necro-inflammation (G0–G4) and staging of the liver fibrosis (S0–S4) were carried out according to Scheuer classification [10] by one experienced pathologist blinded to the clinical data. In the study,Significant fibrosis was defined as fibrosis stage≥S2;Severe fibrosis was defined as fibrosis stage≥S3;Cirrhosis was defined as fibrosis stage = S4.

### Serum markers and noninvasive models

All patients systematically underwent complete biochemical workups, ultrasonography and liver biopsy within 2 days.Blood samples of the subjects were obtained before LB. Biochemical tests were performed by commercial assays in our hospital laboratory for alanine aminotransferase(ALT,U/L), aspartate aminotransferase(AST,U/L), hemoglobin (HGB, g/L), uric acid (UA,μmol/L), Fasting plasma glucose(FPG, mmol/L),Total cholesterol (TC,mmol/L),and Glycerin three greases (TG,mmol/L), high-density lipoprotein (HDL, mmol/L); low-density lipoprotein (LDL, mmol/L). The serum HBV-DNA level was detected with a Real-Time polymerase chain reaction (PCR) System (ABI7700;Applied Shenzhen city Daeran Biological Engineering Co Ltd, Shenzhen, Guangdong,CHN). HBsAg, HBsAb, HBeAg, HBeAb, HBcAb, anti-HCV were measured with CLIA systems(Abbott ARCHITECT i2000 SR system, Abbott Laboratories, Abbott Park, IL, USA).

The formulas of FIB-4, APRI,and AAR were calculated as described in the original articles[4–6].FIB -4:(age [year]*AST [U/L]) / {(PLT [10^9^/L])*(ALT [U/L])^1/2^};APRI:(AST/ [ULN]/PLT [10^9^/L]) *100; AAR:AST(U/L)/ALT(U/L).

### Standardisation of AUROC according to the prevalence of fibrosis stages

It had been found that the prevalence of liver fibrosis stages may be a major factor of variability in assessing the diagnostic accuracy of noninvasive model.Therefore, AUROC should be adjusted according to the prevalence of fibrosis stages using the Difference between advanced and nonadvanced fibrosis (DANA) [11].DANA was calculated according to the following formula:[(prevalence F2*2 + prevalence F3*3 + prevalence F4*4) ⁄ (prevalence F2 + prevalence F3 + prevalence F4)]–[prevalence F1⁄ (prevalence F0 + prevalence F1)]. The adjusted AUROCs (adjAUROCs) were calculated as follows:AdjAUROC = observed AUROC (obAUROC) +0.1056 *(2.5 –DANA).

### Statistical analysis

Continuous data were expressed as mean±SD or median(quartile range)depending on the normality of the data. Continuous variables were compared with one-way ANOVA analysis of variance or Kruskal-Wallis H test, depending on the normality of the data; Categorical variables were expressed as proportions and compared with Chi-square test.

Receiver-operating characteristic (ROC) curves were constructed and the area under the ROC curve(AUROC) were calculated. The overall diagnostic accuracy of different models was evaluated by AUROC. The AUROC values of these models were compared by DeLong’s test[11].

The optimal cut off value was determined by maximal sum of sensitivity and specificity. To further evaluate the clinical utility,the sensitivity (Se), specificity(Sp), positive predictive value (PPV), and negative predictive value (NPV) were calculated using the ROC curve.

To validate diagnostic accuracy and clinical utility of three models, we conducted an internal validation test using bootstrap resampling method. This involved generating ROC curves by drawing 1543 new samples with replacements from the original samples. Then, the AUROCs,sensitivity, specificity, PPV, and NPV accord to the optimal cut off value were calculated in the validation group consisting of 1543 new samples again.

Statistical analyses were performed using SPSS 13.0(SPSS Inc., Chicago, IL).*P* < 0.05 was considered statistically significant.

## Results

### Baselines characteristics of Patients

A total of 1543 patients were recruited into the study with a mean age of 31.55±9.73 years. Of all subjects in the study,1182(76.60%) were male and 361(23.40%) were female, 1168(75.70%) were HBeAg positive and 375(24.30%) were HBeAg negative. The fibrosis stages were 267 (17.30%) in S1, 554 (35.90%) in S2, 423(27.41%) in S3 and 299 (19.38%) in S4. The inflammation grades were 76 (4.93%) in G1, 742 (48.09%) in G2, 527(34.15%) in G3 and 198 (12.83%) in G4. The baseline characteristics were summarized in Table 1. The mean values of FIB-4 and APRI were significantly higher for each successive fibrosis stage (*P* <0.05). There were no differences between successive fibrosis stages for AAR (*p* >0.05,except for S1 VS S2-4 *P* = 0.037).

**Table 1:** Baseline characteristics of 1543 patients with HBV infection.

Parameters	Totel	S1	S2	S3	S4	Test	*P*
	(n = 1543)	(n = 267)	(n = 554)	(n = 423)	(n = 299)	value	value
**Male(n,%)**	1182(76.60)	199(74.51)	417(75.27)	312(73.76)	254(84.95)	14.72	<0.001
**Age(years)**	31.55±9.73	29.43±9.09	30.55±9.23	31.22±9.11	35.79±10.80	70.23	0.002
**ALT(U/L)**	85(48,166)	69(41,128)	90(50,162)	97(48,188)	73(45,167)	21.457	<0.001
**AST(U/L)**	60(42,97)	52(37,71)	58(41,92)	72(48,110)	60(44,108)	54.978	<0.001
**GGT(U/L)**	54(30,99)	32(19,61)	45(27,82)	60(38,113)	84(50,141)	187.99	<0.001
**ALB(G/L)**	44.24±5.42	45.77±4.15	45.16±5.35	43.78±5.62	41.85±5.4	123.94	<0.001
**GLO(G/L)**	27.84±5.01	26.81±4.37	27.18±4.97	28.32±4.75	29.32±5.53	55.68	<0.001
**TBil(umol/l)**	17.55±11.43	16.34±8.44	16.00±10.74	17.96±12.97	20.92±12.21	54.13	<0.001
**DBil(umol/l)**	7.36±8.25	5.83±4.83	6.24±6.21	7.89±9.78	10.08±10.6	77.89	<0.001
**PT(seconds)**	12.17±1.95	11.86±2.05	12.01±1.46	12.11±2.22	12.83±2.1	146.19	<0.001
**WBC(G/L)**	5.81±1.65	6.03±1.63	5.83±1.59	5.79±1.67	5.6±1.73	9.46	0.024
**HGB(G/L)**	143.12±18.79	143.20±21.52	145.52±18.37	142.05±18.83	140.09±16.26	26.53	<0.001
**PLT(G/L)**	188.68±56.87	203.75±52.84	200.44±56.16	186.88±52.71	155.99±54.03	155.18	<0.001
**BUN(umol/l)**	4.22±1.26	4.37±1.29	4.23±1.24	4.11±1.22	4.24±1.32	8.29	0.04
**Cr(umol/l)**	79.15±23.77	78.78±19.96	78.14±20.02	78.41±18.33	82.93±36.55	2.35	0.503
**Glu(mmol/l)**	4.52±1.24	4.43±0.94	4.51±1.14	4.52±1.2	4.65±1.65	0.76	0.860
**TC(mmol/l)**	3.91±1.76	3.89±2.00	4.04±1.75	3.82±1.73	3.83±1.57	13.16	0.004
**TG(mmol/l)**	1.00±0.66	0.94±0.61	1.04±0.75	0.97±0.61	1.02±0.6	3.27	0.352
**LogDNA(copies/ml)**	5.87±1.40	6.12±1.43	5.97±1.4	5.77±1.39	5.6±1.36	29.87	<0.001
**HBeAg+(n,%)**	1168(75.70)	206(77.22)	444(80.15)	323(76.33)	195(65.16)	13.89	<0.001
**Antiviral therapy**	169(10.95)	39(14.6)	61(11.1)	38(9.0)	31(10.4)	5.44	0.142
**G1(n,%)**	76(4.93)	52(19.48)	23(4.15)	0(0)	1(0.33)	181.6	<0.001
**G2(n,%)**	742(48.09)	207(77.53)	417(75.27)	104(24.59)	14(4.68)		
**G3(n,%)**	527(34.15)	7(2.62)	111(20.04)	284(67.14)	125(41.81)		
**G4(n,%)**	198(12.83)	1(0.37)	3(0.54)	35(8.27)	159(53.18)		
**AAR**	0.70(0.49,1.09)	0.69(0.50,1.03)	0.65(0.45,1.0)	0.69(0.49,1.12)	0.79(0.57,1.25)	20.444	<0.001
**APRI**	0.87(0.56,1.42)	0.63(0.44,0.97)	0.78(0.50,1.27)	1.01(0.65,1.59)	1.09(0.73,1.89)	129.68	<0.001
**FIB-4**	1.12(0.74,1.82)	0.87(0.62,1.23)	0.98(0.65,1.52)	1.20(0.80,1.95)	1.70(1.12,2.99)	183.38	<0.001

Footnotes:Hepatic steatosis were diagnosed by liver biopsy. Continuous data were expressed as mean±SD or median(quartile range)and compared with one-way ANOVA analysis of variance or Kruskal-Wallis H test, depending on the normality of the data. Categorical variables were expressed as proportions and compared with Chi-square test.ALT, Alanine aminotransferase;AST, Aspartate aminotransferase;γ-GT, γ-glutamyl transferase; hemoglobin,HGB;UA, uric acid;FPG, Fasting plasma glucose;TC, Total cholesterol;TG, Triglyceride;HDL, high-density lipoprotein cholesterol; LDL, low-density lipoprotein cholesterol; FIB-4,the fibrosis index based on the four factors; APRI, aspartate aminotransferase -to-platelet ratio index;AAR,aspartate aminotransferase–alanine aminotransferase ratio index.

### Diagnostic accuracy of noninvasive models for prediction of fibrosis

**Fig 1:**
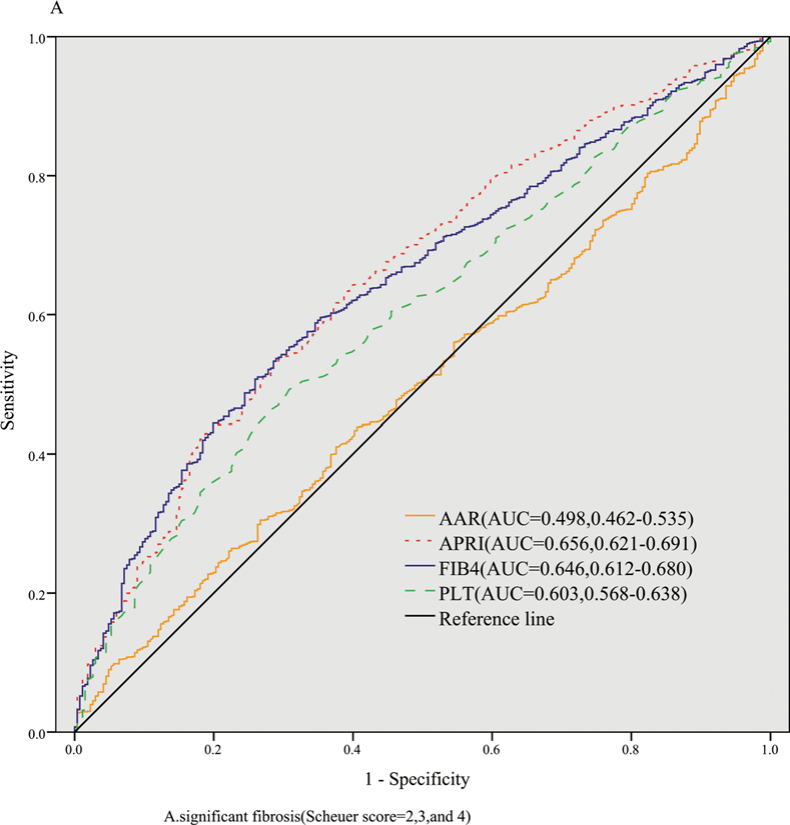
The diagnostic accuracy of noninvasive models for predicting significant fibrosis.

**Fig 2:**
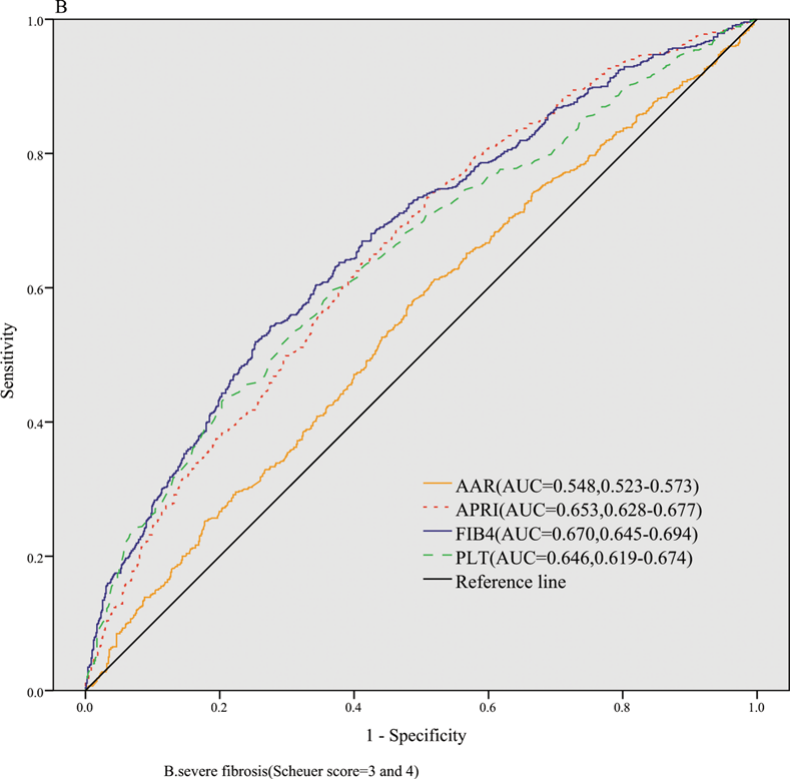
The diagnostic accuracy of noninvasive models for predicting severe fibrosis.

**Fig 3:**
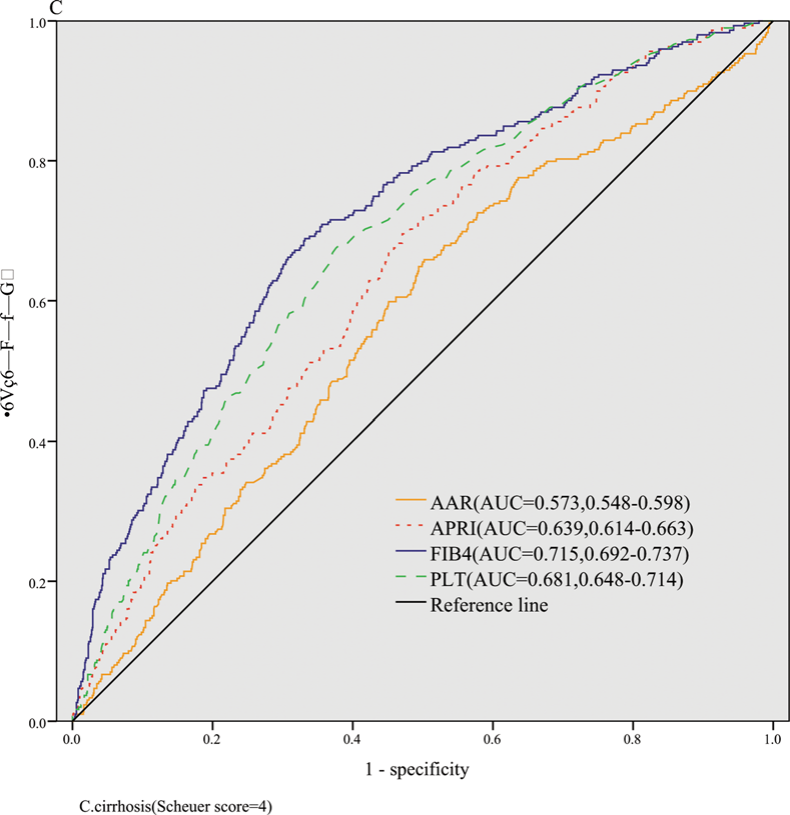
The diagnostic accuracy of noninvasive models for predicting cirrhosis.

The AUROCs of FIB-4, APRI, AAR,and PLT for identification of significant fibrosis were 0.646(ADjAUROC 0.717,95%CI: 0.612–0.680),0.656(ADjAUROC 0.727,95%CI: 0.621–0.691), 0.498(ADjAUROC 0.569,95%CI: 0.462–0.535),and 0.603(ADjAUROC 0.674,95%CI: 0.568–0.638), respectively (Fig 1). The AUROCs of FIB-4, APRI, AAR,and PLT for severe fibrosis were 0.670(ADjAUROC 0.741,95%CI: 0.646–0.694), 0.653(ADjAUROC 0.724,95%CI: 0.628–0.677), 0.548(ADjAUROC 0.619,95%CI: 0.523–0.573), and 0.646(ADjAUROC 0.717,95%CI: 0.619–0.674), respectively (Fig 2). The AUROCs of FIB-4, APRI, AAR,and PLT for cirrhosis were 0.715(ADjAUROC 0.786,95%CI: 0.692–0.737), 0.639(ADjAUROC 0.710,95%CI: 0.614–0.663), 0.573(ADjAUROC 0.644,95%CI: 0.548–0.598),and 0.681(ADjAUROC 0.752,95%CI: 0.648–0.714), respectively (Fig 3).

Then we conducted the comparisons of AUROCs among different tests by DeLong’s test[12]. There were no significant differences of AUROCs between FIB-4 and APRI (*P* = 0.505) for predicting significant fibrosis,which were both superior to AAR (all *P* < 0.01). To predict severe fibrosis, FIB-4 and APRI had same diagnostic accuracy (*P* = 0.170), while the AUROCs of FIB-4 and APRI were better than that of AAR(all *P* < 0.001). FIB-4 was superior to APRI (*P* < 0.001) and APRI was superior to AAR(*P* = 0.008) in predicting cirrhosis.

### Clinical utility of FIB-4,APRI and AAR for prediction of fibrosis

To explore the clinical utility of these models for prediction of fibrosis, The optimal cut off value was determined by maximal sum of sensitivity and specificity. The sensitivity, specificity, PPV, and NPV were summarized in Table 2.

**Table 2:** Clinical utility of three models for prediction of fibrosis.

model	Cut-off	sensitivity	95%CI	specificity	95%CI	PPV	95%CI	NPV	95%CI
	value	%		%		%		%	
**S1 vs S2-4**									
**FIB-4**	1.13	53.3	50.5–56.1	71.4	65.6–76.8	89.9	87.5–92.0	24.2	21.3–27.4
**APRI**	0.72	63.8	61.0–66.4	60.5	54.4–66.4	88.5	86.3–90.5	25.9	22.5–29.5
**AAR**	0.41	17.1	15.1–19.3	88.0	83.5–91.7	87.2	82.4–91.1	18.2	16.1–20.4
**S1-2 vs S3-4**									
**FIB-4**	1.32	54.3	50.6–58.0	72.4	69.2–75.4	63.4	59.4–67.2	64.3	61.1–67.4
**APRI**	0.69	74	70.7–77.2	48.4	44.9–51.9	55.8	52.6–59.0	67.9	64.0–71.7
**AAR**	0.66	58.7	55.0–62.3	50.1	46.6–53.6	50.9	47.4–54.3	58.0	54.2–61.6
**S1-3 vs S4**									
**FIB-4**	1.35	68.9	63.3–74.1	66.8	64.1–69.4	33.4	29.7–37.3	89.9	87.8–91.8
**APRI**	0.84	69.6	64.0–74.7	52.8	50.0–55.6	26.2	23.2–29.4	87.8	85.2–90.1
**AAR**	0.66	65.9	60.2–71.2	48.8	46.0–51.7	23.6	20.8–26.7	85.6	82.8–88.1

Footnotes: Cut-off value was determined by maximal sum of sensitivity and specificity.PPV,positive predictive value;NPV,negative predictive value;CI, confidence interval.

### Validation of models using bootstrap resampling method

To validate the diagnostic accuracy and clinical utility of these noninvasive models for prediction of fibrosis, we conducted an internal validation test using bootstrap resampling method.

There was a good agreement in diagnostic accuracy and clinical utility between the results obtained from the original samples and the bootstrap samples(Table 3). In validation group,there was no significant difference of AUROCs between FIB-4 and APRI (*P* = 0.841) for predicting significant fibrosis,which were both superior to AAR (all *P* < 0.01). To predict severe fibrosis, FIB-4 and APRI had same diagnostic accuracy (*P* = 0.283), while the AUROCs of FIB-4 and APRI were better than that of AAR(all *P* < 0.001). FIB-4 was superior to APRI (*P* < 0.001) and APRI was superior to AAR(*P* = 0.007) in predicting cirrhosis.

**Table 3:** Diagnostic accuracy and clinical utility for prediction of fibrosis in validation group.

model	AUROC	95%CI	ADj	Cut-off	sensitivity	95%CI	specificity	95%CI	PPV	95%CI	NPV	95%CI
			AUROC	value	%	%	%	%	%	%	%	%
**S1 vs S2-4**												
**FIB-4**	0.627	0.603–0.652	0.698	1.13	52.0	48.8–54.4	69.5	63.9–74.7	87.9	85.3–90.1	25.2	22.2–28.3
**APRI**	0.631	0.606–0.655	0.702	0.72	63.7	61.0–66.4	57.9	52.0–63.6	86.6	84.2–88.8	27.2	23.7–30.9
**AAR**	0.502	0.477–0.527	0.573	0.41	84.1	81.9–86.1	12.3	8.8–16.7	80.4	78.2–82.5	15.3	11.0–20.6
**S1-2 vs S3-4**												
**FIB-4**	0.665	0.641–0.689	0.736	1.32	53.2	49.4–57.0	72.9	69.8–75.9	61.3	57.3–65.2	65.9	62.8–68.9
**APRI**	0.652	0.627–0.676	0.723	0.69	74.6	71.1–77.8	48.3	44.9–51.7	53.8	50.5–57.0	70.2	66.3–73.9
**AAR**	0.540	0.515–0.565	0.611	0.66	56.0	52.2–59.7	51.5	48.1–54.9	48.1	44.6–51.7	59.2	55.6–62.8
**S1-3 vs S4**												
**FIB-4**	0.720	0.696–0.742	0.791	1.35	67.3	61.5–72.6	69.2	66.6–71.8	33.3	29.5–37.3	90.2	88.2–92.0
**APRI**	0.640	0.616–0.664	0.711	0.84	67.6	61.8–73.0	54.0	51.2–56.8	25.2	22.1–28.4	87.9	85.4–90.1
**AAR**	0.572	0.547–0.597	0.643	0.66	63.1	57.2–68.7	50.7	47.9–53.5	22.6	19.8–25.7	85.7	83.0–88.2

Footnotes: Cut-off value was determined by maximal sum of sensitivity and specificity.PPV,positive predictive value;NPV,negative predictive value;CI,confidence interval.

### Subgroup analysis of diagnostic accuracy for patients with normal ALT and elevated ALT

To assess the diagnostic accuracy and clinical utility of these noninvasive models for patients with normal ALTand elevated ALT,the datum of patients were separated by normal ALT(defined as ALT<40U/L) and elevated ALT(defined as ALT≥40U/L).The baseline characteristics of patients with normal ALT and elevated ALT were summarized in Table 4.

**Table 4:** Baseline characteristics of patients with normal ALT and elevated ALT.

	ALT<40U/L	ALT≥40U/L	Test value	*P* value
**n**	280	1263		
**Male(n,%)**	195(69.60)	987(78.15)	9.25	0.002
**Age(years)**	32.11±10.79	31.43±9.48	0.981	0.327
**ALT(U/L)**	26(19,33)	157(65,188)	-28.18	<0.001
**AST(U/L)**	43(28,49)	98(48,108)	-16.29	<0.001
**GGT(U/L)**	47(18,56)	84(34,109)	-9.93	<0.001
**ALB(G/L)**	44.22±5.04	44.24±5.50	-0.76	0.939
**GLO(G/L)**	27.03±5.02	28.02±4.99	-2.99	0.003
**TBil(umol/l)**	16.17±10.25	17.85±11.66	-2.23	0.026
**DBil(umol/l)**	6.20±5.45	8.73±7.62	-3.478	<0.001
**PT(seconds)**	11.92±2.37	12.22±1.84	-2.41	0.016
**WBC(G/L)**	5.77±1.75	5.82±1.63	-0.487	0.627
**HGB(G/L)**	139.6±22.63	143.9±17.75	-2.976	0.003
**PLT(G/L)**	180.4±57.78	190.5±56.53	-2.699	0.007
**BUN(umol/l)**	4.33±1.27	4.20±1.26	1.576	0.115
**Cr(umol/l)**	77.60±19.20	79.50±24.66	-1.206	0.228
**TC(mmol/l)**	3.83±1.85	3.93±1.74	-0.908	0.364
**TG(mmol/l)**	0.92±0.63	1.02±0.67	-2.158	0.031
**LogDNA(copies/ml)**	5.42±1.62	5.97±1.33	-5.244	<0.001
**HBeAg+(n,%)**	200(71.43)	931(73.71)	0.61	0.434
**G1(n,%)**	34(12.14)	42(3.33)	44.42	<0.001
**G2(n,%)**	143(51.07)	599(47.43)		
**G3(n,%)**	74(26.43)	453(35.87)		
**G4(n,%)**	29(10.36)	169(13.38)		
**S1(n,%)**	62(22.14)	205(16.23)	7.26	0.064
**S2(n,%)**	101(36.07)	453(35.87)		
**S3(n,%)**	64(22.86)	359(28.42)		
**S4(n,%)**	53(18.930	246(19.48)		
**AAR**	2.04(1.03,2.20)	0.77(0.45,0.84)	8.487	<0.001
**APRI**	0.66(0.37,.0.80)	1.51(0.62,1.53)	-8.974	<0.001
**FIB-4**	1.79(0.85,2.14)	1.56(0.73,1.76)	1.724	0.085

The AUROCs of FIB-4 for patients with normal ALT and elevated ALT were 0.698 and 0.642 for significant fibrosis, 0.702 and 0.670 for severe fibrosis,0.772 and 0.704 for cirrhosis respectively. The AUROCs of APRI for patients with normal ALT and elevated ALT were 0.679 and 0.646 for significant fibrosis, 0.713 and 0.645 for severe fibrosis,0.744 and 0.630 for cirrhosis respectively (Table 5).

**Table 5:** AUROC and ADjAUROC for patients with normal ALT and elevated ALT.

	ALT<40U/L			ALT≥40U/L		
	AUROC	95%CI	ADj AUROC	AUROC	95%CI	ADj AUROC
**n**	280			1263		
**S1 vs S2-4**						
**FIB-4**	0.698	0.628–0.767	0.769	0.642	0.603–0.681	0.713
**APRI**	0.679	0.609–0.749	0.75	0.646	0.606–0.685	0.717
**AAR**	0.580	0.500–0.661	0.651	0.512	0.472–0.552	0.583
**PLT**	0.621	0.547–0.694	0.692	0.603	0.563–0.643	0.674
**S1-2 vs S3-4**						
**FIB-4**	0.702	0.640–0.765	0.773	0.670	0.640–0.699	0.741
**APRI**	0.713	0.651–0.775	0.784	0.645	0.614–0.675	0.716
**AAR**	0.556	0.488–0.624	0.627	0.573	0.542–0.605	0.644
**PLT**	0.656	0.590–0.722	0.727	0.648	0.617–0.678	0.719
**S1-3 vs S4**						
**FIB-4**	0.772	0.709–0.836	0.843	0.704	0.667–0.740	0.775
**APRI**	0.744	0.678–0.811	0.815	0.630	0.592–0.669	0.701
**AAR**	0.614	0.536–0.692	0.685	0.588	0.547–0.628	0.659
**PLT**	0.752	0.684–0.820	0.823	0.676	0.639–0.713	0.747

## Discussion

The results of the present study showed that the AUROCs of FIB-4 were 0.646,0.670 and 0.715 for prediction of significant fibrosis,severe fibrosis,and cirrhosis,while it were 0.656,0.653 and 0.639 for APRI respceively. After standardisation according to the prevalence of fibrosis stages, ADjAUROCs of FIB-4 were 0.717,0.741 and 0.786 for prediction of significant fibrosis,severe fibrosis,and cirrhosis,while it were 0.727,0.724 and 0.710 for APRI respceively.The comparisons of AUROCs in the original group and validation group confirmed that FIB-4 and APRI had similar diagnostic accuracy in predicting significant and severe fibrosis,while FIB-4 was superior to APRI in predicting cirrhosis. Subgroup analysis demonstrated that the diagnostic accuracy of FIB-4 and APRI in patients with normal ALT were higher than that in patients with elevated ALT.

The major conclusions of our study were consistent with that of three previous meta analysis studies. Xu et al. reported that the areas under the SROC curve of FIB-4 and APRI were 0.75 and 0.77 for significant fibrosis, while it were 0.87 and 0.75 for cirrhosis, respectively[13].Li et al. reported that AUROCs of FIB-4 and APRI were 0.78 and0.79 for significant fibrosis, while it were 0.89 and 0.75 for cirrhosis, respectively[14]. Xiao et al. reported that the summary AUROC values of FIB-4 and APRI were 0.78 and 0.74 for significant fibrosis, 0.82 and 0.73 for severe fibrosis, 0.84 and 0.73 for cirrhosis respectively[15].These results demonstrated that the diagnostic accuracy of FIB-4 was similar to that of APRI for significant fibrosis while FIB-4 was superior to APRI in predicting cirrhosis.

The original AUROCs of FIB-4 and APRI in our study seemed to be lower than that of some previous studies, whereas the ADjAUROCs of FIB-4 and APRI in our study were similar to that of previous studies. Omer Basar et al. found that AUROCs of FIB-4 and APRI were 0.741 and 0.669 for significant fibrosis, 0.738 and 0.681 for severe fibrosis, 0.768 and 0.741 for cirrhosis[16]. V. MALLET et al. showed that AUROCs were 0.810 and 0.730 for FIB-4 and APRI in predicting fibrosis [17]. Fatma Ucar et al. reported that AUROCs were 0.687 and 0.662 for FIB-4 and APRI to predict fibrosis[18]. H. Wang et al. found that AUROCs of FIB-4 and APRI were 0.770 and 0.770 in predicting significant fibrosis, 0.810 and 0.770 for severe fibrosis [19]. Beom Kyung Kim et al. reported that AUROCs of FIB-4 and APRI were 0.910 and 0.702 for severe fibrosis, 0.926 and 0.731 in predicting cirrhosis [20]. Jing Ma et al. reported that AUROCs of FIB-4 and APRI were 0.789 and 0.731 for predicting severe fibrosis, 0.804 and 0.740 to predict cirrhosis[21].

On the other hand, similar results to our study were observed in some previous studies,showing lower diagnostic accuracy of FIB-4 and APRI for fibrosis[22–25].In the original study, Sterling et al.reported that the AUROC of FIB-4 in the training and validation cohorts were 0.711 and 0.688 for significant fibrosis, 0.737 and 0.765 for advanced fibrosis[22].Sebastiani et al. found that AUROCs of APRI and FIB-4 were 0.68(0.62–0.74) and 0.66(0.61–0.71) for significant fibrosis in 2411 patients with chronic liver disease;further analysis showed that AUROC s of APRI was 0.64(0.58–0.70) for significant fibrosis and 0.61(0.55–0.66) for cirrhosis in HBV patient[8].Wai et al.reported that the AUROC of APRI were 0.63(0.55–0.71) for significant fibrosis and 0.64(0.54–0.71) for cirrhosis[23].Bonnard P et al.reported that the AUROC of APRI and FIB-4 were 0.61(0.46–0.76) and 0.71(0.57–0.84) for significant fibrosis,0.50(0.32–0.68) and 0.74(0.60–0.87) for cirrhosis [24].

The disagreement between our study and previous studies may be correlated to several potential reasons. First, the heterogeneity may affect the results in different studies. Xu et al. found that the heterogeneity of APRI for detecting significant fibrosis was affected by median age, and for cirrhosis was affected by etiology[13]. Li et al.found that the potential influential factors of heterogeneity were mean age of subjects, prevalence of fibrosis stages, disease spectrum, a consecutive or random sample enrollment, interval between noninvasive model and liver biopsy, the liver blinded biopsy interpretation and a predefined cutoff value[14].Second, it had been found that the prevalence of liver fibrosis stages may be a major factor of variability and a cause of unsatisfactory results in assessing the diagnostic accuracy of noninvasive model.Therefore, the original AUROC should be adjusted according to the prevalence of fibrosis stages for further comparisons [11]. After calibration for prevalence of fibrosis stages,the ADjAUROCs of FIB-4 and APRI in our study were similar to that of previous studies. Third,the mean age of patients in our study was 31.55 years,which was younger than that of most previous studies and may impacted the results of the current study.Fourth, scoring systems of liver pathological diagnosis were different in these studies,affecting directly the results of the studies. The effect of different scoring system must be take into account while preforming comparisons of diagnostic accuracy between different studies. Fifth, sample size was important to construct a convincing conclusion for assessment of diagnostic accuracy. Some previous studies performed analysis base on a relatively small sample size,which might reduce the convince of the conclusions

The results of subgroup analysis showed that diagnostic accuracy of FIB-4 and APRI in patients with normal ALT were higher than that in patients with elevated ALT.Wang et al. reported that the AUROCs for patients with normal ALT was 0.81 for FIB-4 and 0.80 for APRI, compared with 0.71 for FIB-4 and 0.72 for APRI in patients with mildly elevated ALT level[19]. Poynard et al. reported that performance of non-invasive biomarkers was in line with that in patients with elevated ALT[25].On the other hand,some studies reported that performance of non-invasive biomarkers may be somewhat reduced in patients with normal ALT[26–29]. Consequently,further research is needed to determine the clinical utility of FIB-4 and APRI in patients with normal ALT.

There were several advantages in the present study. First,this study had a large sample size,which could reduce the sampling error and conduct a more convincing conclusion. Second, to enhance the credibility of results, we performed an internal validation to confirm the results of the present study by means of bootstrap resampling analysis with replacement.This method was proposed for internal validation of surgical regression models[30].The main advantage of this method is that the original samples can be used to build a more robust model,which can be used to assess the diagnostic accuracy[31].Third,the previous studies assessed the diagnostic accuracy of the FIB-4,APRI,and AAR for significant fibrosis and cirrhosis,but few studies evaluated and compared the diagnostic accuracy for severe fibrosis. For a more comprehensive understanding of the diagnostic accuracy for fibrosis,we attempted to explore the diagnostic accuracy and clinical utility for significant fibrosis,severe fibrosis,and cirrhosis.

There were two limitations in our study.First,all patients in this study were recruited from department of infectious diseases of The Shunde First People’s Hospital,which may reduce the representative of the study population.We recommend that future clinical studies should base on a large scale multi-center population to further compare the diagnostic accuracy and clinical utility of these models for hepatic steatosis in patients with HBV infection.Second,as a retrospective study, some important indicators such asα2-macroglobulin and ferritin could not obtain in the study.

In conclusion,the current study showed that FIB-4 and APRI have similar diagnostic accuracy in predicting significant fibrosis and severe fibrosis,while FIB-4 is superior to APRI for prediction of cirrhosis. The clinical utility of FIB-4 need further external validation in larger population before it was used in predicting fibrosis in patients with HBV infection.
